# The predictive value of suPAR for glomerular segmental sclerosis lesions in renal pathology

**DOI:** 10.1080/0886022X.2025.2498628

**Published:** 2025-05-13

**Authors:** Zurong Zhang, Chongbin Liu, Yachun Han, Daming Li, Yao Huang, Kewen Shi, Shiyu Xia, Jinying Wei, Hong Liu, Lin Sun, Xuejing Zhu, Shuguang Yuan

**Affiliations:** a Department of Nephrology, The Second Xiangya Hospital of Central South University, Changsha, China; b Hunan Key Laboratory of Kidney Disease and Blood Purification, Changsha, China; c Department of Laboratory Medicine, The Second Xiangya Hospital of Central South University, Changsha, China

**Keywords:** suPAR, IgAN, FSGS, Renal pathology

## Abstract

**Introduction:**

Soluble urokinase-type plasminogen activator receptor (suPAR) is a potential circulating pathogenic factor in idiopathic focal segmental glomerulosclerosis (iFSGS); however, its role remains controversial. Glomerular segmental sclerosis lesions (S lesions) are commonly observed in IgA nephropathy (IgAN). This study aims to investigate the correlation between suPAR and S lesions, particularly in IgAN, and to evaluate the potential of suPAR as a prognostic marker.

**Methods:**

Patients were selected from the Second Xiangya Hospital, including 47 cases of IgAN with S lesions (IgAN(S1 group)), 13 cases of IgAN without S lesions (IgAN(S0 group)), and 13 cases of iFSGS. Healthy control group of 20 individuals was selected. Serum suPAR concentration was measured, and its correlation with clinical and pathological data was analyzed. Patients were followed to evaluate the prognostic value of suPAR.

**Results:**

Compared to the healthy control group and IgAN (S0 group), patients in the iFSGS and IgAN (S1 group) had significantly higher serum suPAR concentration (*p* < 0.05). In IgAN patients, serum suPAR concentration was negatively correlated with eGFR (r = −0.458, *p* < 0.05) and positively correlated with the presence and proportion of S lesions (*r* = 0.448, *p* < 0.001; *r* = 0.435, *p* < 0.05). The incidence of composite kidney endpoint events was significantly higher in IgAN patients with high baseline serum suPAR concentration than in those with low level of suPAR (*p* < 0.05).

**Conclusions:**

High serum suPAR concentration is a predictor of S lesions in the kidney and a risk factor for poor prognosis, making it a potential therapeutic target and prognostic predictor for kidney disease.

## 1. Introduction

Urokinase-type plasminogen activator receptor (uPAR) is a membrane-anchored protein encoded by *PlauR* that is present on the surface of immune cells, podocytes, and other cell types [1]. uPAR promotes plasminogen activation and plays a crucial role in biological processes such as cell migration, invasion, and extracellular matrix remodeling [2]. Under stimuli such as inflammation, uPAR detaches from the cell surface to form the soluble urokinase-type plasminogen activator receptor (suPAR) [2]. Focal segmental glomerulosclerosis (FSGS) is classified into four types: idiopathic, secondary, genetic, and of unknown etiology. Studies have shown that suPAR is a potential circulating pathogenic factor in idiopathic focal segmental glomerulosclerosis (iFSGS). It mediates podocyte foot process effacement by binding to and activating podocyte αvβ3 integrin, ultimately leading to the development of proteinuria and the progression of iFSGS [3]. However, the role of suPAR in secondary focal segmental glomerulosclerosis remains unclear.

Secondary focal segmental glomerulosclerosis is commonly associated with IgA nephropathy (IgAN), which is the most prevalent primary glomerular disease worldwide [4]. IgAN is a major cause of end-stage renal disease (ESRD), with approximately 50–75% of IgAN patients progressing to ESRD within 20 years of diagnosis [5]. The Oxford classification for IgAN (assessing the five lesions M, E, S, T, and C, abbreviated as MEST-C) is commonly used for assessing the severity of IgAN [6,7]. Among these classifications, glomerular segmental sclerosis lesions (S lesions) are similar to those observed in iFSGS and are closely related to patient prognosis [8–10]. Patients with S lesions tend to have worse outcomes; however, there is currently a lack of specific serological monitoring indicators for this condition [11]. Guo et al. have suggested that plasma suPAR is a potential predictor of S lesions in patients with IgAN [12]. In contrast, Zhao et al. found no correlation between plasma suPAR level and S lesions in IgAN, but they observed a positive correlation between plasma suPAR and the degree of podocyte foot process effacement [13]. Furthermore, some studies have indicated that suPAR does not cause podocyte damage *in vitro* or *in vivo* [14]. Additionally, these studies did not explore the relationship between circulating suPAR level and the proportion of S lesions in IgAN. Therefore, the role of suPAR in S lesions associated with IgAN warrants further investigation.

Our study aims to investigate the relationship between serum suPAR concentrations and S lesions in kidney tissue among patients with different kidney diseases, particularly IgAN, and to evaluate its potential as both a therapeutic ­target and a prognostic marker.

## 2. Study population, materials, and methods

### 2.1. Study population

**Figure 1.**
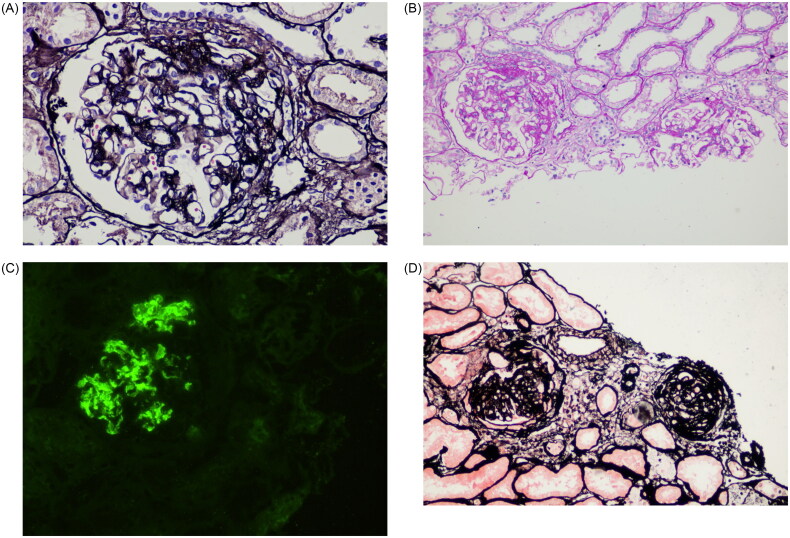
Typical pathological features of iFSGS and IgAN. **(A–B**) Renal pathological changes in iFSGS: (**A**) S lesions in iFSGS patients (PASM, ×200); (**B**) S lesions in iFSGS patients (PAS, ×100). (**C–D**) Renal pathological changes in IgAN: (**C**) Immunofluorescence staining showing strong IgA deposition in the mesangial region of the glomerulus; (**D**) The glomerulus on the left exhibits S lesions in IgAN patients (PASM, ×100).

S lesions are characterized by sclerosis affecting less than 50% of glomeruli (focal) and involving less than 50% of the glomerular lobules (segmental). After excluding secondary and genetic factors, this condition is diagnosed as iFSGS (Figure 1A and B). Among the secondary causes of S lesions, IgAN is one of the most common etiologies. The pathological features of IgAN include IgA deposition in the mesangial region of the glomeruli (Figure 1C), accompanied by mesangial cell proliferation and expansion of the mesangial matrix. While mesangial proliferative IgAN is the predominant pathological subtype, S lesions are also frequently observed (Figure 1D).

In this study, we classified IgAN with S lesions as IgAN (S1 group), and IgAN without S lesions as IgAN (S0 group). The case group included 47 patients with IgAN (S1 group), 13 with IgAN (S0 group), and 13 with iFSGS. The inclusion criteria for the case group were as follows: (1) patients aged 18 years or older at the time of renal biopsy; and (2) patients diagnosed with IgAN (S0 group), IgAN (S1 group), or iFSGS confirmed by renal biopsy at the Second Xiangya Hospital from 2017 to 2022 and verified by two nephropathologists. Exclusion criteria included secondary glomerular diseases, as well as hereditary kidney diseases. Patients with significant dysfunction of vital organs, and those with infections were also excluded. The healthy control group consisted of adults who underwent health examinations at the Second Xiangya Hospital, including 10 healthy adult males and 10 healthy adult females.

### 2.2. Collection of clinical and pathological data

Demographic, clinical, and renal pathological data of the study participants were collected.

### 2.3. Serum sample collection and measurement

On the day of renal biopsy, 2 mL of peripheral venous blood was collected from the patients, allowed to sit at room temperature for 2 h, and then centrifuged at 2000 g for 10 min. The upper serum layer was stored at −80 °C for later use. Serum samples from the healthy control group were obtained from the blood samples collected at the health examination center using the same method. Serum suPAR concentration were measured using the Human uPAR Quantikine ELISA Kit (R&D Systems, Catalog: DUP00).

### 2.4. Statistical analysis

Statistical analyses were performed using SPSS Statistics version 25.0. (IBM Corp, Armonk, NY). Descriptive statistics for continuous variables were presented as mean (standard deviation) or median (interquartile range), while categorical variables were expressed as rates or proportions. For continuous variables that met the assumptions of normality and homogeneity of variance, statistical analyses were performed using t-tests (for two groups) or ANOVA (for more than two groups). For non-normally distributed data, the Mann-Whitney U test (for two groups) or the Kruskal-Wallis test (for more than two groups) was used. Categorical data comparisons were conducted using the chi-squared test or Fisher’s exact test. Correlation analyses were performed using the Pearson or Spearman correlation coefficients. Survival analysis was conducted using the Kaplan-Meier method, and differences between groups were compared using the Log-rank test. Subsequently, univariable Cox proportional hazards regression was performed to estimate hazard ratio (HR) and 95% confidence interval (CI). To assess the robustness of the study results, we conducted a sensitivity analysis. First, we performed multivariable linear regression analysis to adjust for potential confounding factors in the correlation analysis and observed any changes in the results. Second, we calculated the E-value to quantify the potential impact of unmeasured confounders on the prognostic analysis, thereby evaluating the robustness of the study conclusions [15]. Statistical significance was set at *p* < 0.05.

## 3. Results

### 3.1. Baseline clinical characteristics

The overall distribution of sex ratio, age distribution, and mean arterial pressure (MAP) level showed no statistically significant differences among the groups (*p* > 0.05). There was a statistically significant difference in hemoglobin level among the groups, with the IgAN (S1 group) being lower than that of the healthy control group (*p* < 0.05). There was a statistically significant difference in serum albumin (ALB) level among the groups, with the iFSGS group exhibiting lower level than the IgAN (S0 group), and both the iFSGS and IgAN (S1 group) had lower level than the healthy control group (*p* < 0.05). There were no statistically significant differences in blood urea nitrogen (BUN), serum creatinine (Scr), uric acid (UA), or estimated glomerular filtration rate (eGFR) among the groups (*p* > 0.05). A statistically significant difference was observed in proteinuria; specifically, the iFSGS group had higher proteinuria level than the IgAN (S0 group) (*p* < 0.05). Additionally, no statistically significant differences were found in the erythrocyte sedimentation rate (ESR) and C-reactive protein (CRP) level among the groups (*p* > 0.05). Further details are provided in Table 1.

**Table 1.** Baseline clinical characteristics (M(P25, P75) or mean(SD)).

	Healthy Control (*n* = 20)	IgAN (S0 Group) (*n* = 13)	IgAN (S1 Group) (*n* = 47)	iFSGS (*n* = 13)	*P* value
Sex (male/female)	10/10	6/7	11/36	7/6	0.056
Age (years)	31.3 (4.7)	31.7 (9.7)	31.0 (25.0, 39.0)	30 (25.5, 52.5)	0.961
MAP (mmHg)	82.5 (5.6)	79.3 (5.8)	83.0 (77.0, 88.0)	86.9 (8.4)	0.143
Hemoglobin (g/L)	140.0 (129.8, 162.8)	132.5 (17.7)	129.3 (16.6)	139.6 (21.3)	0.007^a^
ALB (g/L)	44.9 (2.5)	42.5 (6.3)	38.9 (36.2, 42.7)	35.1 (21.9, 40.0)	<0.001^b^
BUN (mmol/L)	4.8 (0.8)	4.0 (3.7, 5.0)	4.5 (3.7, 5.5)	6.1 (2.2)	0.114
UA (umol/L)	303.1 (48.2)	305.8 (83.6)	296.4 (241.3, 339.0)	390.9 (125.3)	0.137
Scr (μmol/L)	68.3 (16.3)	65.9 (17.7)	66.0 (57.3, 83.4)	71.9 (47.8, 92.0)	0.834
eGFR(mL/min/1.73 m^2^)	116.2 (108.3, 121.8)	116.7 (110.3, 126.4)	111.5 (95.0, 122.5)	104.6 (30.7)	0.325
Proteinuria (mg/d)	/	145.3 (67.2, 246.6)	525.2 (218.5, 849.2)	1,305.7 (444.9, 3,800.2)	0.001^c^
ESR (mm/h)	/	10.2 (4.3)	13.5 (8.0, 22.5)	21.4 (17.3)	0.242
CRP (mg/l)	/	1.8 (1.2, 2.7)	1.9 (1.3, 2.4)	1.9 (0.9)	0.833

**a.** There was a statistically significant difference in hemoglobin level between the IgAN(S1 group) and healthy control group (adj. *p* = 0.005). **b.** There were statistically significant differences in the overall distribution of serum albumin level between the iFSGS group and the IgAN (S0 group), as well as between the iFSGS group, IgAN (S1 group), and the healthy control group, with adj. *p* = 0.009, adj. *p* < 0.001, and adj. *p* < 0.001, respectively. **c.** There was a statistically significant difference in the overall distribution of proteinuria between the iFSGS and IgAN (S0 group) (adj. *p* = 0.001). MAP, mean arterial pressure; ALB, albumin; BUN, blood urea nitrogen; Scr, serum creatinine; UA, uric acid; eGFR, estimated glomerular filtration rate; ESR, erythrocyte sedimentation rate; CRP, C-reactive protein.

### 3.2. Comparison of serum suPAR concentration

**Figure 2.**
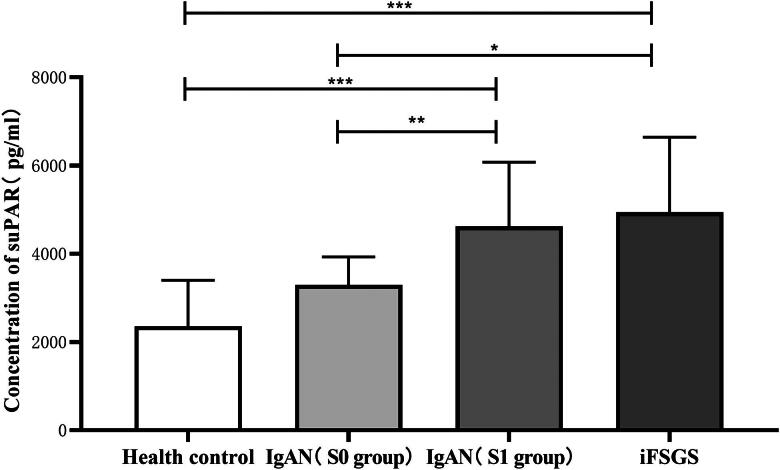
Comparison of serum suPAR concentration. *p = 0.036, **p = 0.016, ***p < 0.001.

The mean (median) serum suPAR concentration in the healthy control group, IgAN (S0 group), IgAN (S1 group), and iFSGS group were 2359.1 (1041.6) pg/ml, 2916.4 (2726.1, 3960.5) pg/ml, 4351.4 (3447.7, 5254.7) pg/ml, and 4944.6 (1699.3) pg/ml, respectively. There was a statistically significant difference in the overall distribution of serum suPAR concentration among the four groups, as shown in Figure 2. Serum suPAR concentration in the IgAN (S1 group) and iFSGS group were higher than those in the healthy control group (*p* < 0.001). Although the serum suPAR concentration in the IgAN (S0 group) was higher than that in the healthy control group, the difference was not statistically significant (*p* = 0.976). The serum suPAR concentration in the iFSGS group and IgAN (S1 group) were higher than in the IgAN (S0 group) (*p* = 0.036, 0.016). Although the serum suPAR concentration in the iFSGS group was higher than that in the IgAN (S1 group), the difference was not statistically significant (*p* = 1.000). The serum suPAR concentration in healthy adult males was 2288.8 (1144.1) pg/ml, while in females, it was 2429.4 (985.1) pg/ml. The concentration in females was slightly higher than that in males, but the difference was not statistically significant (*p* = 0.772).

### 3.3. Linear correlation analysis of serum suPAR concentration with clinical indicators

**Figure 3.**
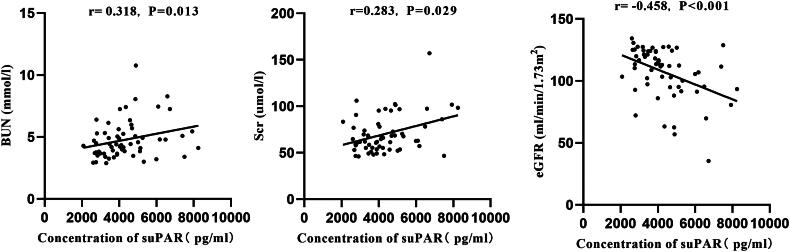
Correlation analysis of suPAR concentration with clinical indicators in patients with IgAN.

The aforementioned studies indicated that serum suPAR concentration is elevated in patients with iFSGS and secondary FSGS (IgAN (S1 group)). To clarify the relationship between serum suPAR concentration and renal function impairment, we analyzed the correlation between serum suPAR level and clinical indicators. First, we assessed the correlation of serum suPAR concentration with various clinical indicators in IgAN patients (Table 2). We found a positive correlation between serum suPAR concentration and Scr and BUN level (*r* = 0.283, *p* = 0.029; *r* = 0.318, *p* = 0.013), while it showed a negative correlation with eGFR level (r = −0.458, *p* < 0.001), as shown in Figure 3. In the cohort of IgAN patients, we did not observe any correlation between suPAR and age, gender, or proteinuria (*p* = 0.416, 0.412, 0.137). Similarly, in the iFSGS group, there was no observed correlation between serum suPAR concentration and age, gender, eGFR, or proteinuria (*p* = 0.552, 0.590, 0.100, 0.627).

**Table 2.** Correlation analysis of suPAR concentration with clinical indicators in IgAN patients.

Clinical Indicators	Correlation Coefficient (r)	*P* value
Sex (male/female)	0.108	0.412
Age (years)	0.107	0.416
MAP (mmHg)	0.158	0.227
White Blood Cell (× 10^9^/L)	0.090	0.495
Hemoglobin (g/L)	−0.083	0.053
Platelet (× 10^9^/L)	−0.004	0.097
ALB (g/L)	−0.115	0.384
Scr (μmol/L)	0.283	0.029
eGFR (mL/min/1.73 m^2^)	−0.458	<0.001
BUN (mmol/L)	0.318	0.013
UA (μmol/L)	0.037	0.780
ESR (mm/h)	0.224	0.118
CRP (mg/L)	−0.153	0.275
Proteinuria (mg/d)	0.213	0.137

MAP, mean arterial pressure; ALB, albumin; BUN, blood urea nitrogen; Scr, serum creatinine; UA, uric acid; eGFR, estimated glomerular filtration rate; ESR, erythrocyte sedimentation rate; CRP, C-reactive protein.

### 3.4. Linear correlation analysis of serum suPAR concentration with pathological indicators in patients with IgAN

The above study indicates that serum suPAR concentration is associated with renal function impairment in patients with IgAN. Subsequently, we conducted a correlation analysis between serum suPAR concentration and pathological indicators in patients with IgAN. The analysis results showed that serum suPAR concentration was positively correlated with S lesions, tubular atrophy/interstitial fibrosis (T lesions), and crescents (C lesions) in the Oxford classification (*r* = 0.448, *p* < 0.001; *r* = 0.389, *p* = 0.002; *r* = 0.279, *p* = 0.031). However, no correlation was observed with mesangial proliferation (M lesions) and endothelial cell proliferation (E lesions) (*p* = 0.301, 0.711).

**Figure 4.**
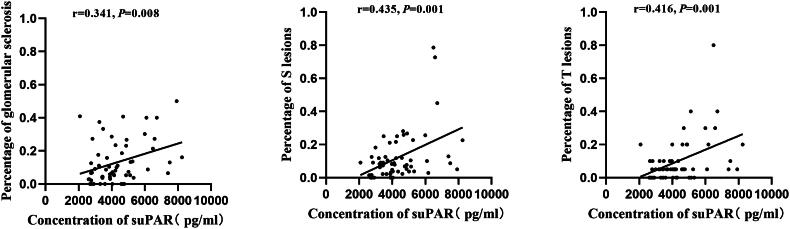
Correlation analysis of suPAR concentration with pathological indicators in patients with IgAN.

Further analysis of the correlation between serum suPAR concentration and the severity of IgAN lesions revealed positive correlations with the proportion of global sclerosis, S lesions, and T lesions (*r* = 0.341, *p* = 0.008; *r* = 0.435, *p* = 0.001; *r* = 0.416, *p* = 0.001), as shown in Figure 4. Additionally, we further analyzed the correlation between serum suPAR concentration and the degree of podocyte foot process effacement, vacuolation, and microvillous change observed under electron microscopy in IgAN patients, but no correlations were found (*p* = 0.324, 0.244, 0.879).

### 3.5. The predictive value of serum suPAR concentration for IgAN with S lesions

**Figure 5.**
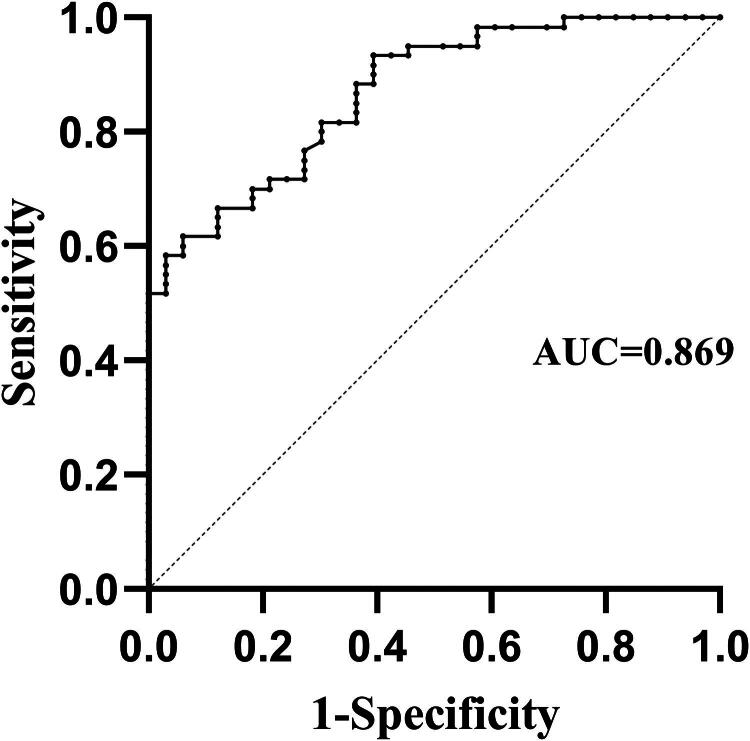
ROC curve of suPAR for predicting S lesions in kidney tissue of IgAN patients.

Studies have shown that S lesions are closely related to the prognosis of patients with IgAN, with those accompanied by S lesions having a worse prognosis [11]. Therefore, determining whether patients with IgAN have S lesions is crucial for the prognostic assessment. In this study, we used the receiver operating characteristic (ROC) curve to evaluate the predictive value of serum suPAR concentration for IgAN with S lesions. The results are shown in Figure 5, with an area under the curve (AUC) of 0.869 ± 0.036 (*p* < 0.001). The optimal cutoff value was 4042.060 pg/ml, with a sensitivity of 61.7% and specificity of 93.9%.

### 3.6. Follow-up study

The above study shows that suPAR is closely related to the presence of S lesions in IgAN. Follow-up was conducted on patients in the IgAN (S1 group), with one patient lost to follow-up, resulting in a total of 46 patients included in the follow-up study. In addition to common renal endpoint events such as ESRD, a sustained decrease in eGFR of ≥ 40%, and renal-related mortality [16], studies have shown that IgAN patients with an eGFR decline slope of ≥ 3 mL/min/1.73 m^2^/year (aged ≤ 40 at diagnosis) have a lifetime risk of renal failure of 100% [17]. In our follow-up study, these four indicators were defined as composite renal endpoint events.

**Figure 6.**
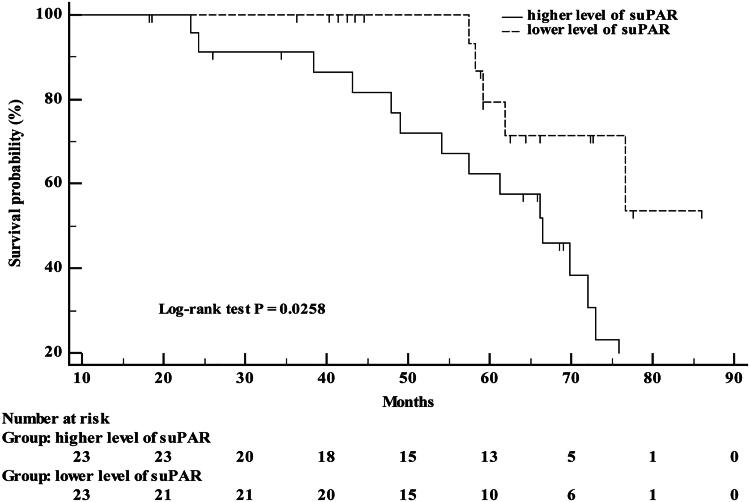
Kaplan-Meier Survival curve for time to composite renal endpoint events in IgAN (S1 group) patients.

Based on the median serum suPAR concentration, patients were divided into a high-level group (23 patients) and a low-level group (23 patients), with a median follow-up time of 60.29 (42.94, 70.34) months. At the end of the follow-up period, 15 patients in the high-level group and 5 patients in the low-level group experienced composite renal endpoint events. Among these, one patient progressed to ESRD and three patients had a sustained decrease in eGFR of ≥ 40%, all from the high-level group. The remaining patients had an eGFR decline slope of ≥3 mL/min/1.73 m^2^/year. Kaplan-Meier survival analysis showed that the incidence of composite renal endpoint events was significantly higher in the high-level group compared to the low-level group, with a statistically significant difference (*p* = 0.0258), as shown in Figure 6. Univariate Cox proportional hazards regression analysis showed that, compared to the low baseline serum suPAR concentration group, the high-level group had a significantly higher incidence of composite renal endpoint events (HR = 3.013, 95% CI: 1.089–8.335, *p* = 0.034).

### 3.7. Sensitivity analyses

Multiple linear regression was performed to further investigate the relationship between serum suPAR concentration and clinical as well as pathological indices in IgAN patients. The analysis included serum suPAR concentration, eGFR, and S, T, and C lesions. The results demonstrated that serum suPAR concentration was positively correlated with S lesions (*p* = 0.029) and negatively correlated with eGFR levels (*p* = 0.027). However, no significant correlation was observed between serum suPAR concentration and T or C lesions (Table 3).

**Table 3.** Multivariate linear regression of suPAR with clinical and pathological indicators in IgAN patients.

	β	SE	t	*P* value
intercept	5467.165	1004.958	5.440	<0.001
eGFR	−18.698	8.230	−2.272	0.027
S lesions	922.871	412.146	2.239	0.029
T lesions	807.357	478.923	1.686	0.098
C lesions	184.936	351.164	0.527	0.601

eGFR, estimated glomerular filtration rate; S lesions, glomerular segmental sclerosis lesions; T lesions, tubular atrophy/interstitial fibrosis; C lesions, crescents.

In prognostic analysis, E-value analysis is used to quantify the potential impact of unmeasured confounders on the observed exposure-outcome association. In this study, it was found that among IgAN (S1 group) patients, the high serum suPAR concentration group had a significantly higher incidence of composite renal endpoint events compared to the low-level group (HR = 3.013, 95% CI: 1.089–8.335, *p* = 0.034). The calculated E-value was 5.476, indicating that only the presence of an unmeasured confounder associated with both the exposure and outcome with an HR of at least 5.476 could fully explain away the observed association.

## 4. Discussion

suPAR is a potential circulating pathogenic factor in iFSGS, but its role in secondary focal segmental glomerulosclerosis remains unclear. Secondary FSGS is commonly observed in IgAN, and both previous studies and our research have confirmed that IgAN patients with S lesions have a poorer prognosis [11,18]. Establishing whether IgAN is associated with S lesions and assessing their severity are crucial for prognostic evaluation. However, currently, there is a lack of specific serum biomarkers to evaluate and monitor the presence and severity of S lesions. Research has shown that S lesions associated with IgAN are similar to those observed in iFSGS [8–10]. To explore the relationship between circulating suPAR and iFSGS as well as S lesions in IgAN, we measured serum suPAR concentration in four groups: healthy control group, IgAN (S0 group), IgAN (S1 group), and iFSGS. We first matched and adjusted for factors that may influence serum suPAR concentration, including gender, age, eGFR, and inflammatory markers ESR and CRP level of the study participants [19–23]. The results showed that serum suPAR concentration in patients with iFSGS and IgAN (S1 group) were higher than those in patients with IgAN (S0 group) and healthy control group, suggesting that suPAR may be associated with the occurrence of iFSGS and S lesions in IgAN. This finding indicates that there may be common mechanisms underlying the pathogenesis of these conditions.

Univariate linear correlation analysis revealed that serum suPAR concentration were negatively correlated with eGFR level in the IgAN cohort, which is consistent with the findings of other studies [12,13]. However, contrary to other studies, we did not observe a correlation between serum suPAR concentration and age, sex, or proteinuria level [12,13]. In the iFSGS cohort, we did not observe a correlation between serum suPAR concentration and age, sex, eGFR, or proteinuria level, which may be related to the small sample size of this group. However, it is noteworthy that our results indicated lower serum albumin level in the IgAN(S1 group) and iFSGS group, suggesting that these patients may have lost more albumin through urine. The lack of correlation between serum suPAR concentration and proteinuria level could potentially be attributed to the small sample size and the heterogeneity of the patient population.

Previous studies have found that suPAR is involved in the occurrence and progression of S and T lesions in patients with iFSGS [3,24]. Our analysis of the relationship between serum suPAR concentration and pathological indicators in patients with IgAN demonstrated a positive correlation between serum suPAR level and the presence of S, T, and C lesions associated with IgAN. Notably, our research revealed that serum suPAR concentration were positively correlated with the proportion of glomerulosclerosis, S lesions, and T lesions in patients with IgAN, indicating a relationship with the severity of these lesions.

Wei et al. demonstrated that suPAR promotes podocyte motility and the disappearance of foot processes by binding to and activating podocyte αvβ3 integrin [25]. In contrast, Harel et al. indicated that suPAR is not a direct cause of podocyte injury [14]. Our results indicate that there is no correlation between suPAR concentration and the degree of foot process effacement. In contrast, Zhao et al. found a positive correlation between plasma suPAR level and the extent of podocyte foot process effacement in IgAN [13]. It remains unclear whether suPAR promotes the occurrence and progression of kidney disease by damaging podocytes. In our study, we did not observe a relationship between serum suPAR concentration and podocyte microvilli changes or vacuolar degeneration. The relationship between suPAR and renal ultrastructural lesions is still not well-defined and requires further investigation.

To further validate the above findings, we subsequently conducted a multivariate linear regression analysis of serum suPAR concentration and clinical and pathological indicators in patients with IgAN. The results indicated that serum suPAR concentration remained positively correlated with S lesions in IgAN and negatively correlated with eGFR level. This further suggests that suPAR may be involved in the development of S lesions in patients with IgAN and could serve as a potential novel therapeutic target for treatment.

Research has shown that baseline suPAR concentration are closely related to the prognosis of kidney diseases, such as iFSGS and acute kidney injury [3,26]. Higher baseline suPAR concentration were associated with an increased risk of renal endpoint events and complications. Additionally, reducing suPAR level can significantly improve renal outcomes in mice with acute kidney injury [26]. ROC curve analysis for predicting renal S lesions based on serum suPAR concentration yielded an AUC of 0.869, demonstrating excellent predictive capability [27]. Our follow-up study indicated that high baseline serum suPAR concentration is a risk factor for disease progression in patients with IgAN. The E-value analysis further strengthened the robustness of the study results. This suggests that baseline suPAR concentration could serve as a potential prognostic predictor for IgAN. Targeting the reduction in circulating suPAR level in patients with high suPAR level may improve their prognosis.

## 5. Limitations

Our study has certain limitations. This was a single-center study with a relatively small sample size, which may not fully represent the overall population. Further research is required to elucidate the specific molecular mechanisms involved.

## 6. Conclusion

In conclusion, our study found that serum suPAR concentration is correlated with the presence of S lesions in patients with iFSGS and IgAN, and it is positively associated with the proportion of S lesions in IgAN. High baseline serum suPAR concentration is a risk factor for poor renal prognosis in patients with IgAN. suPAR is involved in the occurrence and development of S lesions, making it a potential target for pharmacological treatment and a prognostic predictor.

## Data Availability

The datasets used and/or analyzed during the current study are available from the corresponding author on reasonable request.
